# Tuning Functional Amyloid Formation Through Disulfide Engineering

**DOI:** 10.3389/fmicb.2020.00944

**Published:** 2020-05-26

**Authors:** Anthony Balistreri, Ethan Kahana, Soorya Janakiraman, Matthew R. Chapman

**Affiliations:** Department of Molecular, Cellular and Developmental Biology, University of Michigan, Ann Arbor, MI, United States

**Keywords:** disulfide engineering, functional amyloid, CsgA, curli, protein engineering

## Abstract

Many organisms produce “functional” amyloid fibers, which are stable protein polymers that serve many roles in cellular biology. Certain Enterobacteriaceae assemble functional amyloid fibers called curli that are the main protein component of the biofilm extracellular matrix. CsgA is the major protein subunit of curli and will rapidly adopt the polymeric amyloid conformation *in vitro*. The rapid and irreversible nature of CsgA amyloid formation makes it challenging to study *in vitro*. Here, we engineered CsgA so that amyloid formation could be tuned to the redox state of the protein. A double cysteine variant of CsgA called CsgA_CC_ was created and characterized for its ability to form amyloid. When kept under oxidizing conditions, CsgA_CC_ did not adopt a β-sheet rich structure or form detectable amyloid-like aggregates. Oxidized CsgA_CC_ remained in a soluble, non-amyloid state for at least 90 days. The addition of reducing agents to CsgA_CC_ resulted in amyloid formation within hours. The amyloid fibers formed by CsgA_CC_ were indistinguishable from the fibers made by CsgA WT. When measured by thioflavin T fluorescence the amyloid formation by CsgA_CC_ in the reduced form displayed the same lag, fast, and plateau phases as CsgA WT. Amyloid formation by CsgA_CC_ could be halted by the addition of oxidizing agents. Therefore, CsgA_CC_ serves as a proof-of-concept for capitalizing on the convertible nature of disulfide bonds to control the aggregation of amyloidogenic proteins.

## Introduction

Amyloids are fibril aggregates of proteins which have misfolded to form a characteristic cross β-sheet secondary structure (Chiti and Dobson, 2006). The fibers deposit in cells in dense bodies known as plaques and are implicated in many well-known neurodegenerative diseases including Alzheimer, Parkinson, and Huntington Disease (Chiti and Dobson, 2017). For this reason, the nature of amyloids and how they form is an active area of research. Though the disease causing amyloids are composed of misfolded proteins, there are now many examples of “functional amyloids” that organisms create for a predetermined purpose (Chapman et al., 2002; Otzen, 2010). These fibers are composed of proteins that adopt the amyloid fold not by misfolding, rather they do so intentionally and efficiently (Deshmukh et al., 2018). Functional amyloids provide researchers with well-defined and adaptable models for studying the basic tenets of amyloid formation.

Curli are functional amyloids produced by *Escherichia coli* and other Enterobacteriaceae (Dueholm et al., 2012). Curli amyloids are the main proteinaceous component of the biofilm extracellular matrix (Chapman et al., 2002; Reichhardt and Cegelski, 2014). The major protein subunit of curli, called CsgA, is secreted in a monomeric, unstructured state (Wang et al., 2007). CsgA undergoes self-assembly into curli fibers on the cell surface with the help of several auxiliary proteins belonging to the *csg* operon (Chapman et al., 2002; Robinson et al., 2006; Hammer et al., 2007; Wang et al., 2008; Nenninger et al., 2009, 2011; Evans et al., 2015; Jain and Chapman, 2019). Purified CsgA readily forms amyloid fibers and there are several methods to monitor *in vitro* amyloid aggregation (Wang et al., 2007; Evans et al., 2018). However, these techniques require handling protein samples which are in a constant state of aggregation (Evans et al., 2018).

To minimize CsgA aggregation into amyloid during purification, the current methodology relies on chemical denaturants (8M guanidinium hydrochloride) (Zhou et al., 2013). Though this protocol provides useable amounts of purified CsgA, there are still several purification steps that need to take place under non-denaturing conditions. During the final size exclusion and buffer exchange steps there is an unavoidable loss of soluble CsgA which is tolerated in exchange for speediness.

Here, we employ a different strategy to restrict amyloid formation. CsgA is an intrinsically disordered protein that transitions into a β-sheet rich conformation upon forming amyloid fibers (Wang et al., 2007). Therefore, a potential strategy for controlling aggregation is targeting this transition. Disulfide bonds are tertiary structural components of many natively folded proteins that aid in protein folding and provide stability (Anfinsen and Scheraga, 1975). They are also by nature convertible; they can be broken and reformed based on the redox state of the environment (Gilbert, 1995). By engineering two cysteine residues into its sequence, we can provide CsgA the option to form an intramolecular disulfide bond under the right conditions. Previous work has shown disulfide engineering can be used to stabilize the native fold state of a protein (Matsumurat et al., 1989; Dombkowski et al., 2014; Schmidt et al., 2015; Liu et al., 2016). Recently, other groups have used this technique to modulate amyloid formation of human amyloid proteins (Hoyer et al., 2008; Carija et al., 2019). In this study we harness disulfide engineering as a method for stabilizing the non-native fold state of a protein with the intention of triggering protein folding.

**FIGURE 1:**
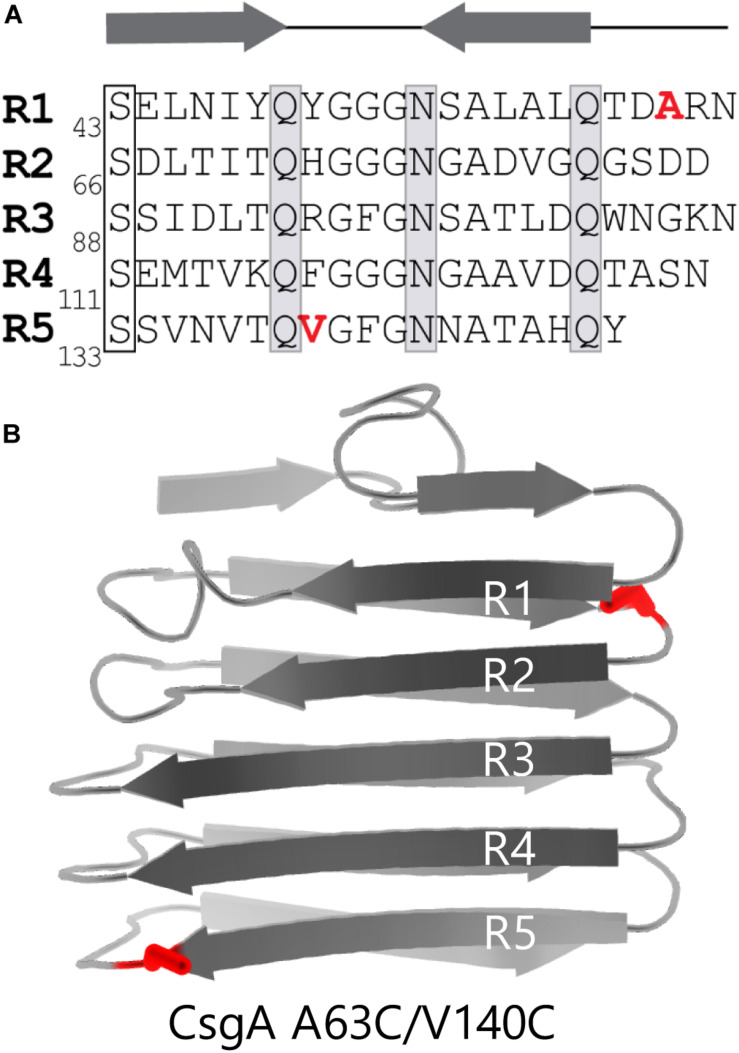
CsgA_CC_ features two cysteine mutations flanking highly amyloidogenic regions of CsgA WT. **(A)** The primary sequence of CsgA contains five imperfect repeats which are labeled R1-R5. Highly conserved serine, glutamine, and asparagine are residues that play a role in amyloid formation are boxed. The location of the two mutated residues are labeled in red. **(B)** Cartoon depiction of a mature and folded CsgA monomer using data from the Lindorff-Larsen lab (Tian et al., 2015). Mutations A63C and V140C are shown in red.

We created a double cysteine variant of CsgA called CsgA_CC_ with the goal of making CsgA amyloid formation tunable to its redox state. CsgA contains five imperfect repeat sequences (R1-R5) corresponding to five β-strands that exist in the final folded protein (Figure 1A; Wang et al., 2007). β-strands R1 and R5 are critical to the ability of CsgA to form amyloid (Wang et al., 2008). We hypothesized that the presence of an unnatural disulfide linkage near R1 and R5 would hinder CsgA from forming the secondary structure required for amyloid formation. In order to allow amyloid formation to eventually occur, regions of the sequence essential to proper folding needed to remain undisturbed. Therefore, the β-turn regions that flank R1 and R5 offered an amenable target region. The sequence of CsgA does not contain any native cysteine residues (Barnhart and Chapman, 2006). We decided to replace two residues of similar size and properties to cysteine in the regions of interest. Alanine-63 and valine-140 fulfilled the criteria and were replaced with cysteine residues (Figure 1B). As we hypothesized, we found that the ability of CsgA_CC_ to form amyloid could be stimulated by the addition of a reducing agent, and that when kept in an oxidized form, CsgA_CC_ remained in a soluble and non-amyloid conformation.

## Materials and Methods

### Bacterial Growth

All overnight cultures were grown in sterilized LB (Fisher Scientific) media supplemented with 100 μg/mL ampicillin or 50 μg/mL of kanamycin at 37°C with shaking at 220 rpm. When necessary, LB agar plates were supplemented with ampicillin 100 μg/mL or kanamycin 50 μg/mL.

### Strains and Plasmids

A full list of strains, plasmids, and primers can be found in Supplementary Tables S1, S2. Site directed mutagenesis was performed on pre-existing plasmids using the Agilent QuikChange II XL kit. Primers were designed by Agilent QuikChange Primer Design (https://www.agilent.com/store/primerDesignProgram.jsp) and synthesized by IDT (https://www.idtdna.com). Mutagenized plasmids were constructed in the MC1061 cell background. Mutations were confirmed using Sanger sequencing done at the UM Biomedical Research Core (https://brcf.medicine.umich.edu/). Plasmids were purified using Promega PureYield^TM^ Plasmid Miniprep System. Miniprep plasmids were transformed into an expression strain (NEB3016), curli-competent strain (MC4100), or a curli-incompetent MC4100 variant strain that lacks *csgA* (LSR10).

### Protein Purification

Recombinant CsgA and its variants were purified as described previously (Evans et al., 2018). An overnight liquid culture was prepared by inoculating 25 mL of LB media supplemented with 25 μL of 100 mg/mL Ampicillin. The culture was grown up overnight in a shaking incubator at 37°C and 220 rpm. The following day two solutions containing 750 mL of LB media and 0.75 mL of 100 mg/mL Ampicillin were inoculated with a 1% inoculum of the overnight liquid culture. The culture was grown up in a shaking incubator at 37°C and 220 rpm until OD600 = 0.8–0.9 was reached. Protein expression was induced by adding 375 μL of 1M ITPG and continue to incubate for 1 h. Induced cells were spun down by centrifugation at 4,000 rpm for 20 min at 4°C and supernatant was discarded. Cell pellets representing 250 mL of culture volume were placed into 50 mL Falcon tube for storage and frozen at –80°C. A frozen cell pellet was retrieved from storage and resuspended in 25 mL of Lysis buffer (8 M guanidinium hydrochloride, 50 mM KPi, pH 7.3) and incubated at room temperature for 1 h on a rocking shaker. The cellular debris was spun down at 10,000 × g for 20 min and the soluble fraction was collected. The soluble fraction was sonicated 3 × 20 s using a probe sonicator. The sonicated mixture was incubated with 0.8 mL of HIS-Select^®^ HF Nickel Affinity Gel (Sigma) and mixed at room temperature for 1 h. The mixture was loaded into a disposable 5 mL polypropylene column containing a fritted glass filter and the flow through was discarded. The column was washed with 10 mL of 50 mM KPi, pH 7.3 and 3 mL of wash buffer (12.5 mM imidazole in 50 mM KPi, pH 7.3), discarding the eluent. 5 mL of elution buffer (125 mM imidazole in 50 mM KPi, pH 7.3) was added to the column and incubated for 5 min at room temperature. The first 0.5 mL of eluent was set aside and the next 1 mL elution fraction was collected. A 2 mL Zeba^TM^ Spin Desalting Column (Pierce) was rinsed twice with 50 mM KPi, pH 7.3 by centrifuging at 1,000 rpm for 2.5 min. The elution fraction was loaded into the column and spun down into a clean 15 mL Falcon tube. The flow through was moved to a cooled Amicon^®^ 30K cut-off filter column and centrifuge at 7,000 × g for 10 min to result in the final solution containing purified protein. After purification, CsgA proteins were stored in 50 mM KPi, pH 7.3 buffer at 4°C unless stated otherwise. Size exclusion chromatography was performed on CsgA_CC_ samples essentially as previously described (Evans et al., 2018). Ni-NTA elution fractions were concentrated using a cooled Amicon^®^ 3K cut-off Spin Column centrifuged at 7,000 rcf for 10 min at 4°C until the total volume was 2̃ mL. When the desired volume was reached, the retentate was moved to MilliporeSigma^TM^ Ultrafree^TM^-MC Centrifugal Filters and centrifuged to remove any particulates. The filtered samples underwent gel filtration using a GE Superdex 75 10/300 GL column attached to an Äkta pure protein purification system and the elution buffer was 50 mM KPi, pH 7.3. FLPC elution fractions were assayed for protein concentration using A220. Samples corresponding to elution peaks were analyzed using reducing (with added β-mercaptoethanol) and non-reducing (without β-mercaptoethanol) SDS-PAGE. CsgC was purified as described previously (Salgado et al., 2011).

### Circular Dichroism (CD)

200 μL of purified protein samples of CsgA WT and CsgA_CC_ were added directly into a quartz cell with a 1 cm path. CD spectra were obtained using a Jasco J–1500 CD Spectrometer by scanning from 190 to 260 nm at 25°C. Where indicated, tris(2-carboxyethyl)phosphine (TCEP) was added from a stock solution (200 mM TCEP in MQ water, pH = 7.7) to a final concentration of 8 mM. The spectrum of the appropriate buffer-only control was subtracted from each sample as a baseline. Figure 3A includes three purification batches Batch 1 (44 μM) Batch 2 (22 μM) and Batch 3 (16 μM) stored at 4°C in 50 mM KP_i_, pH 7.3. For the spectra shown in Figure 3B, 20 μM CsgA WT was stored at room temperature in 20 mM KP_i_, pH 7.3 that contained 25 μM PMSF. Finally, Figure 3C includes 20 μM CsgA_CC_ was that stored at 4°C in 50 mM KP_i_, pH 7.3.

### Denaturing Gel Electrophoresis and Western Blot

Purified protein samples were diluted in 2X SDS loading buffer (1 mL 1.25 M Tris base pH 6.8, 1 mL β-mercaptoethanol, 200 μL 1% bromophenol blue, 2 mL glycerol, 6 mL 10% SDS and 10 mL water) with or without β-mercaptoethanol and run on a 15% SDS PAGE gel (Evans et al., 2018). The gels were microwaved in 30 mL of Coomassie blue dye for 20 s and destained with MQ water overnight to visualize protein bands. Western Blot were performed as previously described using the wet transfer method (Evans et al., 2018). Briefly, blots were probed with a primary antibody against CsgA (1:12,000). Secondary antibodies against rabbit IgG and conjugated with IRDye 800CW were used to image the blots in a LI-COR Odyssey^®^ Fc.

### Gel Solubility Assay

Freshly purified samples of CsgA WT and CsgA_CC_ were transferred to a microcentrifuge tube, diluted to 20 μM in 50 mM KPi pH = 7.3, and allowed to incubate at room temperature. Where indicated, tris(2-carboxyethyl)phosphine (TCEP) was added from a stock solution (200 mM TCEP in MQ water, pH = 7.7) to a final concentration of 8 mM. Gel samples were removed at 0, 24, and 48 h after purification. The gel samples were diluted in 4X SDS loading buffer and run on a 15% SDS PAGE gel (Evans et al., 2018). The gels were stained with Coomassie blue dye. Destained gels were scanned using the 700 nM channel of a LI-COR Odyssey^®^ Fc. Band intensity was quantified using ImageJ (https://imagej.nih.gov/ij/) and normalized to the CsgA WT time 0 no TCEP added condition.

### Transmission Electron Microscopy

TEM images were produced as previously described (Jain et al., 2017). Briefly, 5 μL of purified protein samples were spotted onto a formvar/carbon 200 mesh copper grid. After a 5 min incubation, grids were spotted with 5 μL of DI water followed by 5 μL of 2% uranyl acetate to provide micrograph image contrast. Grids were imaged using a Jeol JEM 1400plus Transmission Electron Microscope.

### Thioflavin T Binding Assay

Th-T assays were performed as previously described (Zhou et al., 2012). Briefly, freshly purified CsgA was diluted with 50 mM KPi, pH 7.3 to 20 μM and combined with 20 μM of the amyloid-specific dye thioflavin-T (Th-T). Selected samples were reduced with the addition of tris(2-carboxyethyl)phosphine (TCEP) from a stock solution (200 mM TCEP in MQ water, pH = 7.7). Amyloid formation was monitored by measuring an increase in Th-T fluorescence at 495 nM (450 nM excitation). Assays were performed in triplicate at microscale within 96-well plates and measured with Tecan infinite^®^ Pro M200 or infinite^®^ Nano^+^ F200 microplate readers. In Figures 4–6, 8 the data were normalized by the largest value of the CsgA WT condition performed at the same time in each experiment. When specified, Th-T assays included the following additions: FN075 diluted from a 50 mM stock solution (DMSO) sourced from the Almqvist Lab (Department of Chemistry, University of Umeå, Sweden), CsgC purified and diluted from a stock solution (phosphate buffer), CsgA WT fibers purified previously and sonicated directly before addition, and hydrogen peroxide.

### Complementation Assay

Congo Red binding assays were performed as previously described (Evans et al., 2018). Overnight liquid cultures were normalized to OD = 1 and a 200 μL aliquot was spun down at 13 k rpm and the supernatant was decanted. The cell pellets were washed by resuspending, pelleting, and decanting the cells in YESCA media (1 g yeast extract, 10 g casamino acids and 20 g agar in 1 L water). After the third wash, the cells were resuspended in 200 μL YESCA and 4 μL was spotted onto a YESCA agar plate supplemented with 50 μg/mL Congo Red. The plates were incubated at 26°C for 48 h to allow for curli formation. After that time plates were photographed to generate the images used in Figure 7A.

## Results

Immediately after purification CsgA is intrinsically disordered with a random coil secondary structure. Within hours, CsgA aggregates into stable, β-rich amyloid fibers (Wang et al., 2007). To control amyloid aggregation by CsgA we hypothesized that strategically-placed cysteine residues would allow the generation of a CsgA molecule that could be locked in a non-amyloid state (Figure 1). The wildtype sequence of *E. coli* CsgA does not contain native cysteine residues (Figure 1A). Cysteine residues were engineered into CsgA at positions alanine-63 and valine-140 using site-directed mutagenesis. The resulting protein, called CsgA_CC_, was purified and characterized. From this point forward native CsgA will be referred to as CsgA WT and CsgA that contains cysteine residues at positions 63 and 140 will be referred to as CsgA_CC_.

**FIGURE 2:**
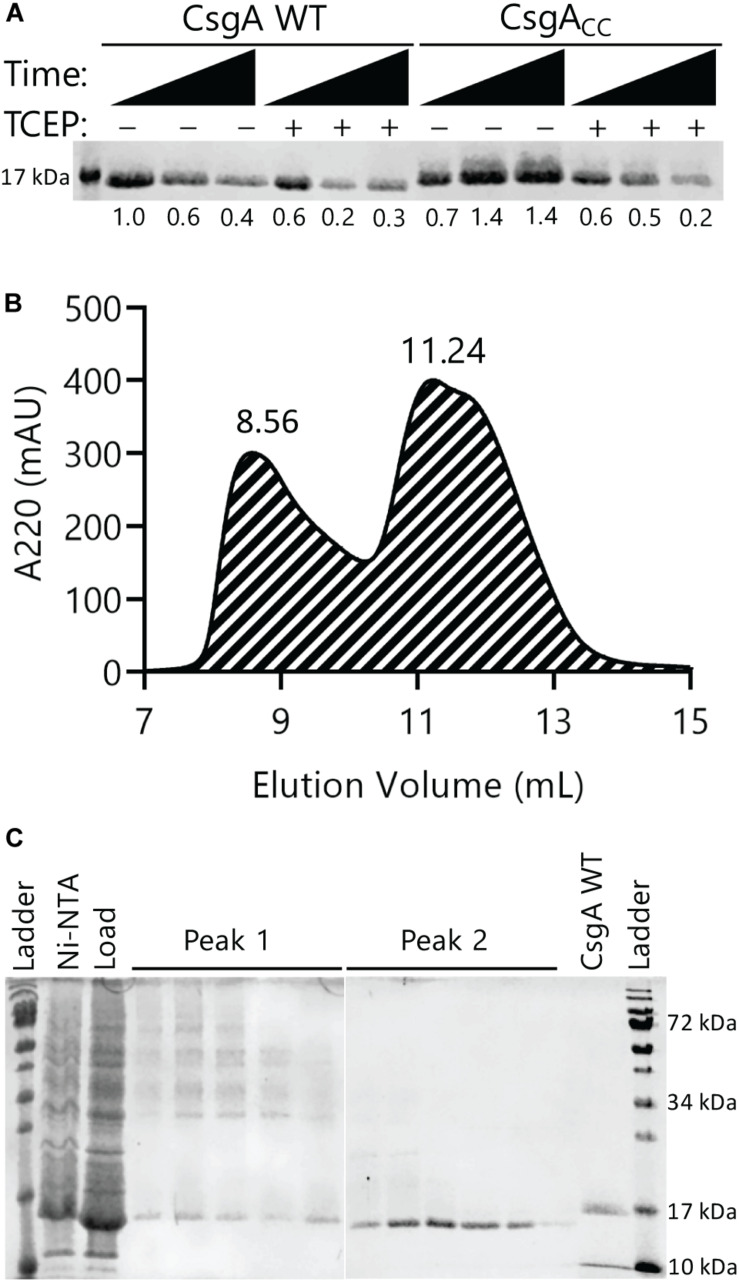
Oxidized CsgA_CC_ remains SDS-soluble and forms predominantly monomeric species. **(A)** Both CsgA WT and CsgA_CC_ were purified and incubated at room temperature in the presence or absence of the reducing agent TCEP. Samples were taken at 0, 24, and 48 h after purification. An SDS-PAGE gel stained with Coomassie blue shows oxidized CsgA_CC_ remains SDS soluble longer than reduced CsgA_CC_ and CsgA WT. The number below each lane represents a quantification of the band intensity normalized to CsgA WT time 0 h with no TCEP added. **(B)** A gel filtration chromatogram showing the elution profile of CsgA_CC_. CsgA_CC_ was purified as described in the Materials and Methods without the addition of a reducing agent. There are two broad peak associated with CsgA_CC_ at 8.56 mL (Peak 1) and at 11.24 mL (Peak 2). **(C)** Non-reducing SDS-PAGE gels that contain the eluents from Peaks 1 and 2 of the size exclusion chromatography experiment.

We found that freshly purified and oxidized CsgA_CC_ remained SDS soluble for at least 48 h (Figure 2A). For comparison, both CsgA WT and CsgA_CC_ that was incubated with the reducing agent TCEP displayed a marked decrease in SDS solubility over a 48 h period (Figure 2A). Because CsgA_CC_ remained soluble, size exclusion chromatography could be used to determine what types of protein species were present after purification. Freshly purified CsgA_CC_ was subjected to gel filtration and the protein eluted off the column in two wide peaks at 8.56 (peak 1) and 11.24 mL (peak 2) elution volume (Figure 2B). Analytical gel filtration chromatography can normally be used to provide an estimate of an analyte’s size by comparing column retention times with known globular proteins in a standard curve (Whitaker, 1963). However, because CsgA_CC_ remained unstructured, the globular proteins did not provide comparable retention profiles (Sambi et al., 2010). We therefore used non-reducing SDS-PAGE analysis to view what size protein species were in solution (Figure 2C). The second and larger peak corresponded to the monomeric species of CsgA_CC_ (Figure 2C). When samples from peak 1 of the gel filtration were analyzed by non-reducing SDS-PAGE, there were several slowly migrating bands (Figure 2C). When the same samples from peak 1 were analyzed by SDS-PAGE that included a reducing agent, β-mercaptoethanol, many of the high molecular weight bands disappeared, suggesting that CsgA_CC_ forms homo-oligomers that disassociate when reduced (Supplementary Figure S1). The monomeric species of CsgA_CC_ incubated under oxidized conditions ran faster on the SDS-PAGE gel than CsgA WT prepared in the same manner (Figure 2C). It is possible that in a non-reducing environment the intramolecular disulfide bond that holds CsgA_CC_ in a constrained conformation results in the faster migration of CsgA_CC_ relative to CsgA WT.

**FIGURE 3:**
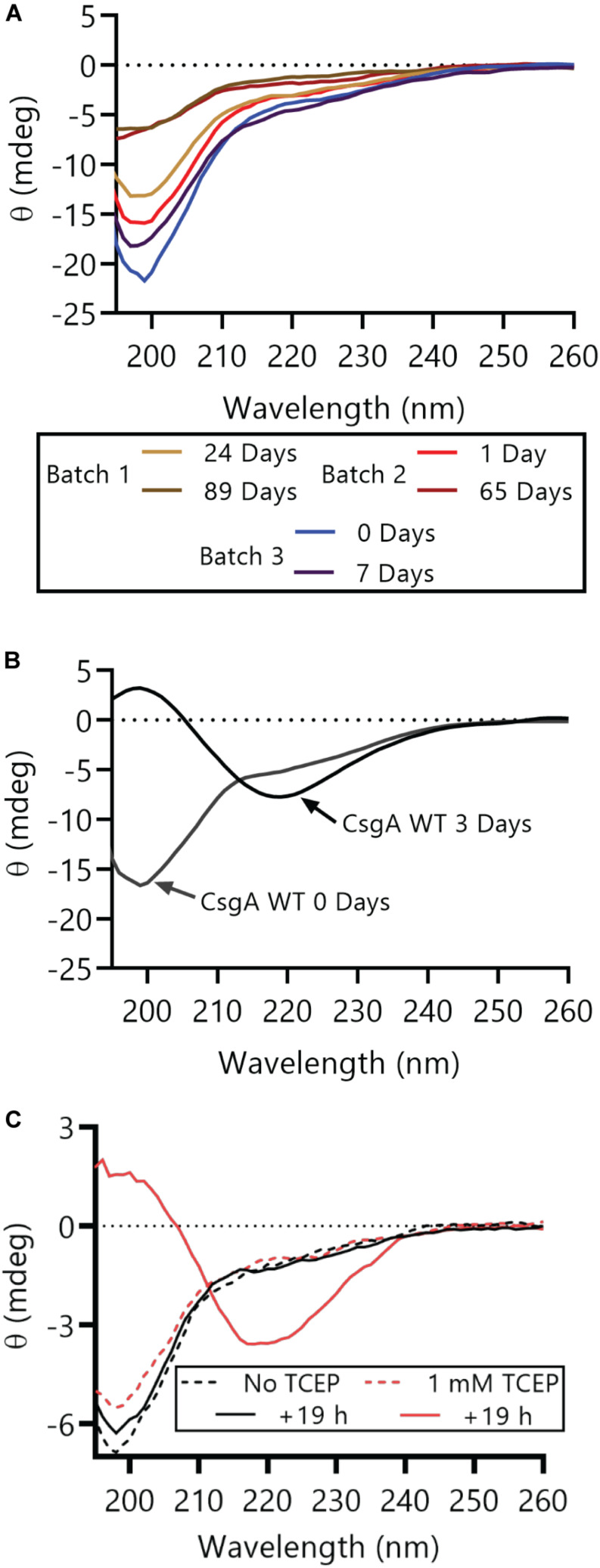
CsgA_CC_ remains random coil secondary structure until treatment with a reducing agent. **(A)** Purified protein samples from three different CsgA_CC_ purification batches were stored at 4°C and their secondary structure was assayed using circular dichroism at the indicated number of days post-purification. **(B)** Purified CsgA WT was stored at room temperature and its secondary structure was assayed at two time points after purification. At day 0 days post-purification CsgA WT had a minimum at around 200 nM and at 3 days post-purification the minimum was around 220 nM. **(C)** Purified CsgA_CC_ that had been incubated at 4°C for 4 days was split into two and incubated at room temperature with or without TCEP for an additional 19 h.

Oxidized CsgA_CC_ can remain in a non-aggregated and non-amyloid form for much longer than CsgA WT. As measured by circular dichroism (CD), CsgA_CC_ remained in a random coil conformation for at least 89 days when stored under oxidizing conditions at 4°C (Figure 3A). CsgA WT transitions from random coil to β-sheet rich in less than 3 days (Figure 3B). When TCEP was added to CsgA_CC_ after 4 days of incubation under oxidizing conditions, CsgA_CC_ transitioned from random coil to β-sheet rich secondary structure (Figure 3C). This suggested that the redox state of CsgA_CC_ is a critical determinant of its secondary structure.

**FIGURE 4:**
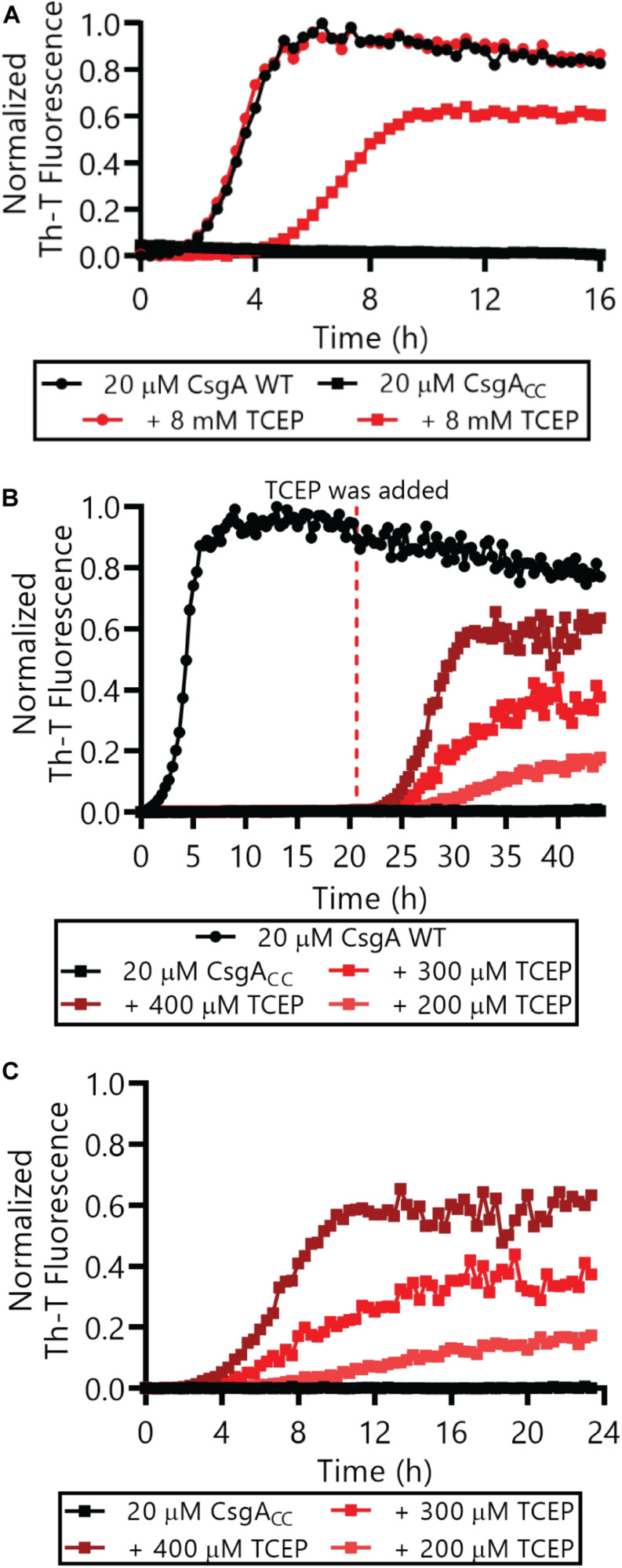
CsgA_CC_ monitored by Th-T fluorescence assays. **(A)** Freshly purified CsgA WT and CsgA_CC_ were monitored by Th-T fluorescence with and without the immediate addition of 8 mM TCEP. **(B)** CsgA WT and CsgA_CC_ amyloid formation was monitored using Th-T fluorescence. TCEP was added to CsgA_CC_ after a 20 h incubation. **(C)** The same data as **(B)** is represented where T = 0 h has been adjusted to reflect the addition of the reducing agent, not the beginning of the experiment. In all panels, the CsgA_CC_ with no reducing agent data are represented by the curves that are mostly superimposed along the x-axis.

**FIGURE 5:**
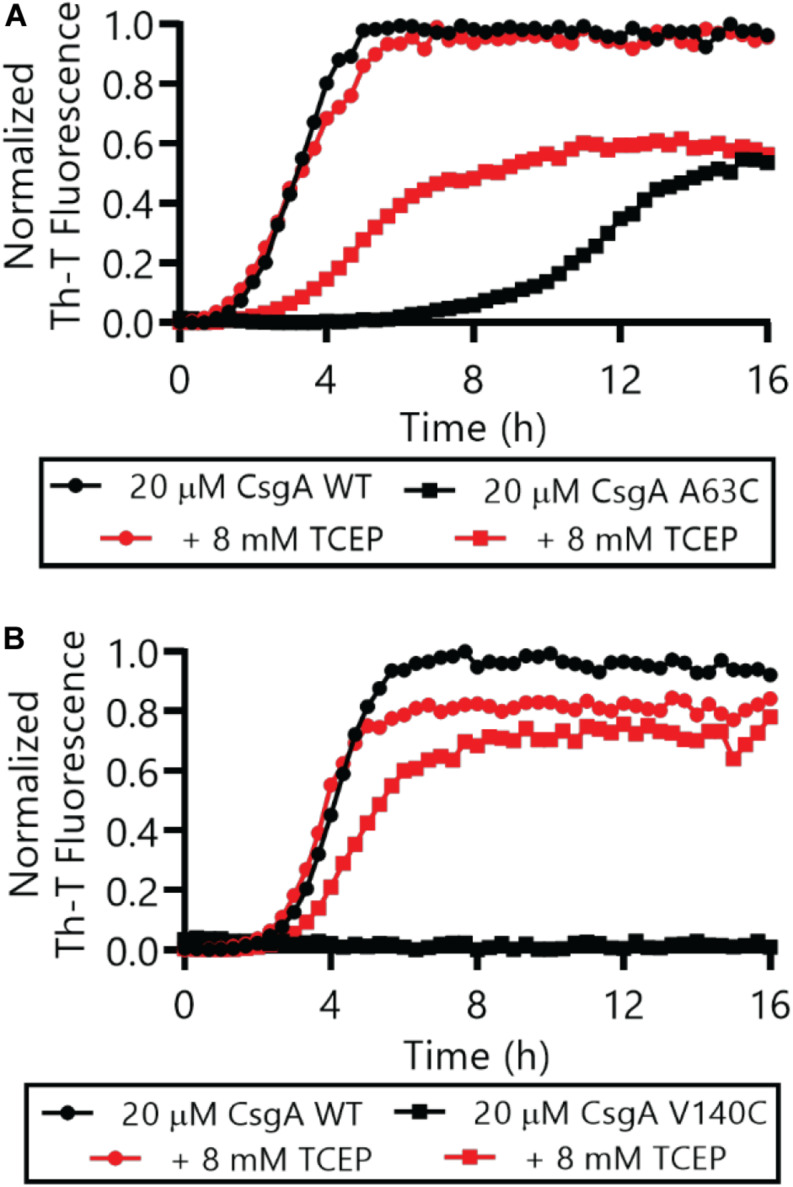
Single cysteine variants CsgA A63C and V140C form amyloid in the absence or presence of a reducing agent. Freshly purified CsgA single cysteine variants A63C **(A)** and V140C **(B)** were monitored by Th-T fluorescence with and without the immediate addition of 8 mM TCEP. The full Th-T curve for V140C can be found in Supplementary Figure S4.

**FIGURE 6:**
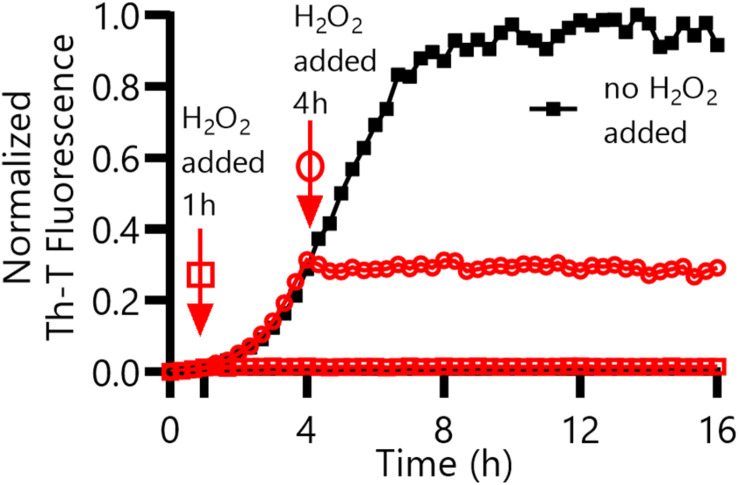
CsgA_CC_ amyloid formation in the presence of an oxidizing agent, H_2_O_2_. CsgA_CC_ was purified and stored at 4°C for 4 days. After 4 days, TCEP was added to each well to a final concentration of 8 mM. Hydrogen peroxide was added to a final concentration of 2% (v/v) after an additional 1 h (square) or 4 h (circle) had elapsed.

The Thioflavin-T (Th-T) binding assay is the most widely used method for monitoring *in vitro* amyloid formation in real time (Nilsson, 2004). CsgA WT amyloid formation displays a characteristic sigmoidal curve during which Th-T fluorescence begins to increase after a 2–4 h lag phase (Wang et al., 2007). Importantly, CsgA_CC_ showed no detectable increase in Th-T fluorescence unless treated with a reducing agent, even after prolonged incubation at room temperature (Figure 4 and Supplementary Figures S2, S3). There was a dose-dependent response of CsgA_CC_ amyloid formation when TCEP was added in a range between 400 and 200 μM (Figures 4B,C). In all experiments there was an observable decrease in overall fluorescence seen in CsgA_CC_ Th-T binding assays when compared to CsgA WT (Figures 4, 8). In order to verify that intramolecular disulfide bonds played a role in inhibiting CsgA_CC_ amyloid formation, we characterized the two single cysteine variants’ ability to form amyloid. The introduction of a single cysteine mutation at either position 63 or 140 was only sufficient to slow down amyloid formation under oxidized conditions. A63C (Figure 5A) and V140C (Figure 5B) formed amyloid with and without the treatment of reducing agent, displaying an extended lag phase when compared to wildtype. Though V140C showed a lag phase that was much longer than A63C (Supplementary Figure S4). It should be noted that the addition of a reducing agent has no effect on the rate of CsgA WT amyloid formation (Figure 5 and Supplementary Figure S2B). In an experiment where CsgA_CC_ amyloid formation had reached a fast growth phase, it could be prematurely stopped after the addition of an oxidizing agent like hydrogen peroxide (Figure 6). These experiments confirmed that CsgA_CC_ amyloid formation is susceptible to change given the redox state of its environment.

**FIGURE 7:**
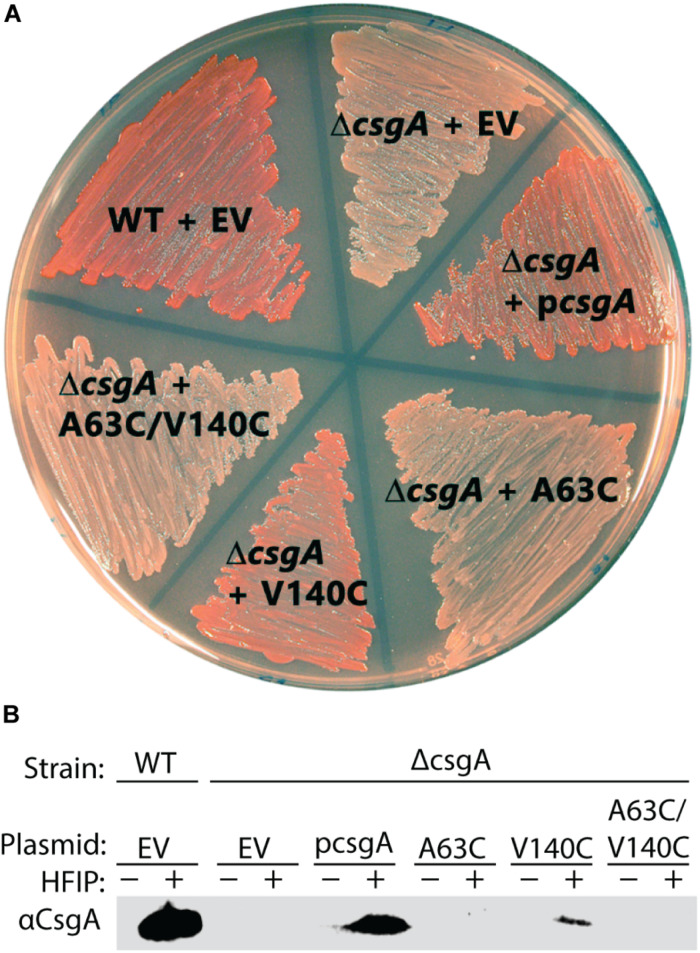
*In vivo* complementation assay shows CsgA_CC_ and CsgA A63C expressing bacteria are unable to form curli. **(A)** A curli competent strain of *E. coli* (MC4100) forms a red colony phenotype when grown on a YESCA plate supplemented with Congo Red. The curli-deficient strains (Δ*csgA)* form a white colony phenotype under the same conditions. The red phenotype can be rescued when Δ*csgA* strains express CsgA WT from a vector (p*csgA*). CsgA_CC_ and CsgA A63C expressing strains cannot rescue the same red phenotype produced by wildtype bacteria. **(B)** Whole cell western blots were performed on cells scraped from the plate above. After treatment with HFIP, a strong denaturant that solubilizes CsgA, monomeric CsgA is only found in the red colored colonies.

The ability of CsgA_CC_ to form amyloid *in vivo* was assessed. Curli-producing *E. coli* display a red colony phenotype when grown on a plate supplemented with the amyloid-binding dye Congo Red (Hammar et al., 1995; Evans et al., 2018). A Δ*csgA* mutant that can no longer produce curli forms white colonies on Congo Red indicator plates (Hammar et al., 1995; Evans et al., 2018). The red colony phenotype can be rescued in a Δ*csgA* strain when CsgA WT is supplied by a plasmid containing the *csgA* gene downstream of the natural promoter (Hammar et al., 1995; Evans et al., 2018; Figure 7A). When Δ*csgA* strains were complemented with a plasmid encoding the variants CsgA A63C, CsgA V140C, and CsgA_CC_ the resulting colonies were white, red, and white, respectively (Figure 7A). Whole cell western blot analysis showed the strain producing V140C was the only strain to harbor protein recognized by a αCsgA antibody (Figure 7B). Therefore, cysteine residues at the 63rd position or at positions 63 and 140 are more disruptive to curli formation than a single cysteine residue in the valine-140 position. The combination of color phenotype and negative western blot results found in the A63C and CsgA_CC_ samples coincided with previously published work describing Δ*csgG* and Δ*csgE* mutants (Robinson et al., 2006; Nenninger et al., 2011). It is possible that CsgA A63C and CsgA_CC_ are confined to the periplasm and subjected to proteolytic degradation in the same manner as CsgA WT in Δ*csgG* and Δ*csgE* mutants (Robinson et al., 2006; Nenninger et al., 2011).

**FIGURE 8:**
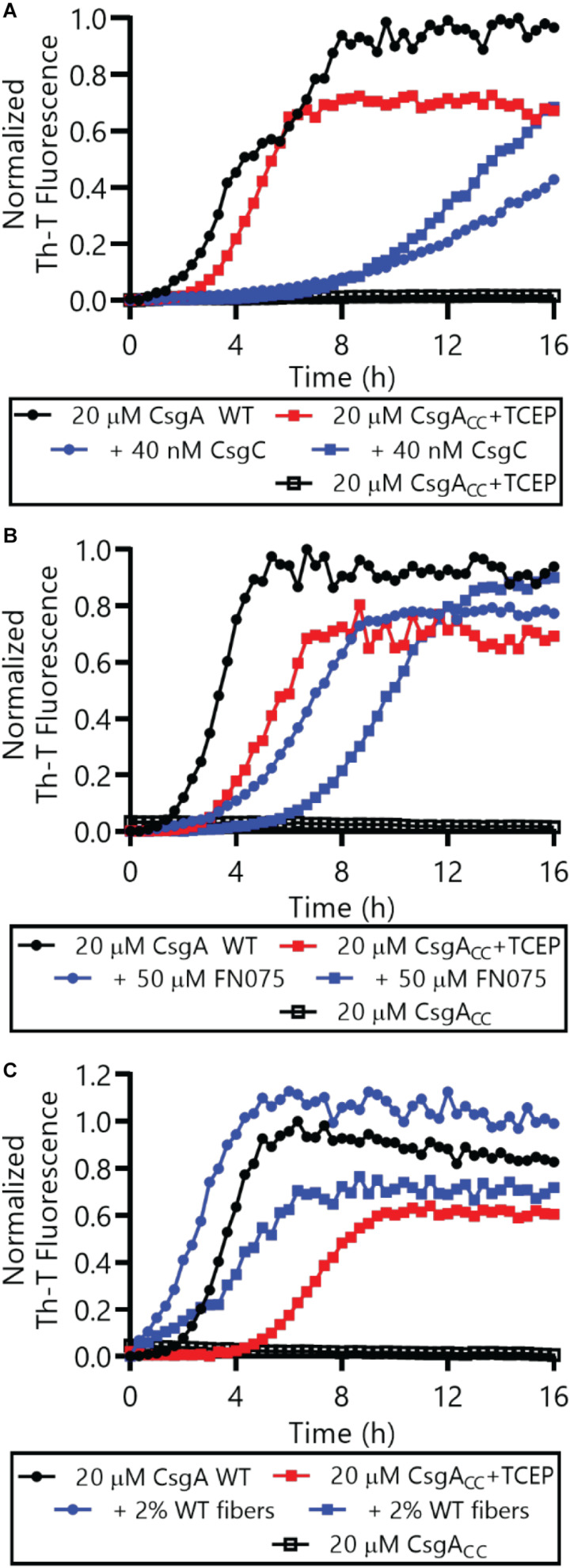
Both CsgA WT and CsgA_CC_+TCEP were incubated in the presence or absence of CsgC **(A)** the 2-pyridone FN075 **(B)**, or 2% WT fibers **(C)** and amyloid formation was monitored by Th-T fluorescence. In all panels, CsgA_CC_ without TCEP are represented by the curves that are mostly superimposed along the x-axis.

**FIGURE 9:**
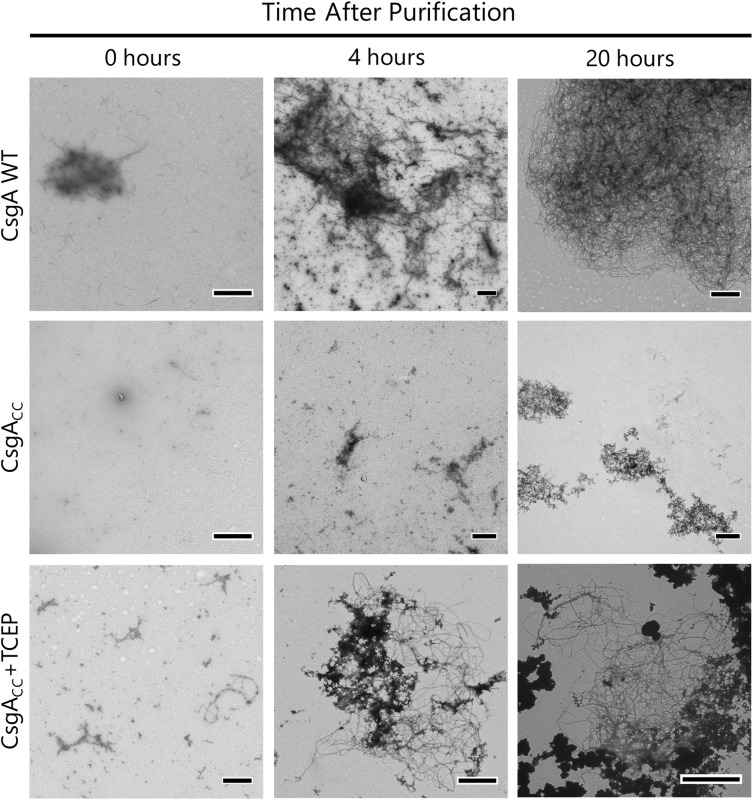
CsgA_CC_+TCEP displays fiber morphology similar to wildtype protein. TEM micrograph showing the mature fiber structures made by CsgA_CC_+TCEP. A 5 μL sample was taken from the well of a Th-T binding assay 0, 4, and 20 h after the addition of a reducing agent. This sample was adsorbed onto a TEM grid and negatively stained with uranyl acetate for contrast. Micrographs shown are representative images of the entire TEM grid. The scale bar is representative of 1 μM.

We also determined whether reduced CsgA_CC_, or CsgA_CC_+TCEP, interacted with certain proteins and small molecules in a similar manner to CsgA WT. Amyloid formation by CsgA WT is inhibited by the CsgC chaperone and also by a 2-pyridone called FN075 (Cegelski et al., 2009; Evans et al., 2015). We incubated CsgA WT and CsgA_CC_+TCEP with inhibitory concentrations of FN075 and CsgC and monitored amyloid formation (Figures 8A,B). Both CsgA WT and CsgA_CC_+TCEP amyloid formation were inhibited *in vitro* with similar efficiencies (Figures 8A,B). Conversely, amyloid formation can be sped up through a “seeding” effect when a small amount of preformed fibers are introduced to freshly purified CsgA WT (Wang et al., 2007). CsgA_CC_+TCEP amyloid formation was similarly sped up when mixed with CsgA WT fibers (Figure 8C). Finally, CsgA_CC_ +TCEP formed fibril structures of identical morphology with wildtype fibers as confirmed by transmission electron microscopy (Figure 9). Fibers could not be found in oxidized CsgA_CC_ samples (Figure 9). This line of evidence suggested that CsgA_CC_, after being reduced, behaves similarly to CsgA WT *in vitro.*

## Discussion

**FIGURE 10:**
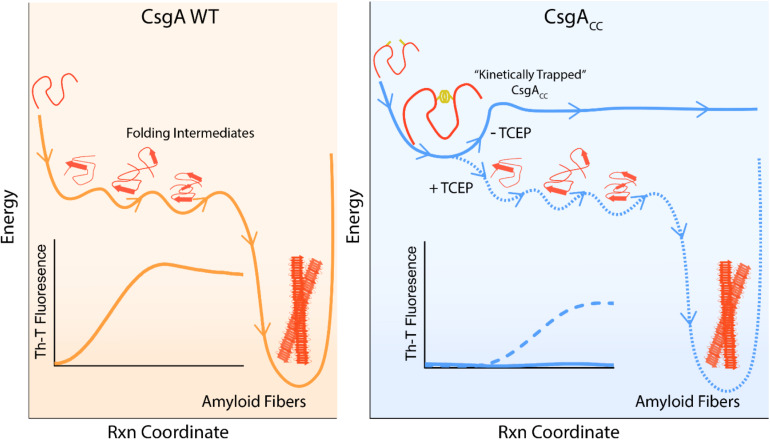
A model depicting CsgA_CC_ as kinetically trapped in a non-amyloidogenic state until it is released by a reducing agent. CsgA WT is an intrinsically disordered protein which initially features random coil secondary structure. CsgA WT eventually folds into a β-sheet rich conformation that is both highly amyloidogenic and energetically favorable. This process can be monitored in ensemble through a Th-T binding assay. On the contrary, CsgA_CC_ remains kinetically trapped in a fold state that is non-amyloidogenic. CsgA_CC_ remains unstructured and monomeric until the disulfide bond is released and the protein is allowed to progress through its normal route toward amyloid formation.

CsgA WT can transition from a mono dispersed, random coil structure to β-rich amyloid fibers within about 3 h (Wang et al., 2007). Indeed, the amyloid fiber conformation represents a very favorable energy minimum for amyloidogenic proteins (Eisenberg and Jucker, 2012). CsgA_CC_ incubated under oxidizing conditions remains in a random coil, non-amyloidogenic state for at least 88 days (Figure 3). The oxidized disulfide bond “kinetically traps” CsgA_CC_ in a fold state of relatively high energy compared to the amyloid conformation into which each protein would naturally deposit (Figure 10). In a reducing environment, the transition of CsgA_CC_ into the amyloid conformation required no additional heat or chemical energy at all (Figure 3B). Moreover CsgA_CC_ exhibits a consistent Th-T lag phase of no shorter than 2–4 h after reduction (Figures 4, 8). Therefore, when CsgA_CC_ is “kinetically trapped” in an oxidized environment, it must be trapped in a way that prevents nucleus formation. The introduction of a nucleating species, such as sonicated fibers, to amyloid-competent proteins provides a dramatic decrease in an energy barrier leading to rapid amyloid formation (Gosal et al., 2005). However, providing oxidized CsgA_CC_ with preformed fibers did not spark amyloid formation (Supplementary Figure S5). CsgA_CC_ transitions from an amyloid-incompetent species to an amyloid-competent species only after the disulfide bond is reduced (Figure 10).

Here, we have shown CsgA_CC_ forms amyloid only when incubated in the presence of a reducing agent (Figures 4, 8), affording a method to tune CsgA_CC_ aggregation depending on its redox state. CsgA_CC_ amyloid formation is sensitive to reducing agent in a dose-dependent manner (Figure 4), thus providing control over when amyloid formation occurs. Because CsgA_CC_ can be kept in a non-amyloidogenic form it should allow for easier purification, handling, and storage of the protein.

CsgA_CC_ could lend itself to understanding the nucleation phase, which is currently poorly understood for functional amyloids like CsgA WT (Arosio et al., 2015). CsgA WT amyloid formation begins directly after purification (Wang et al., 2007; Zhou et al., 2013). By the time we start to monitor CsgA WT amyloid formation by Th-T fluorescence, aggregation and nuclei formation has already started (Sleutel et al., 2017). On the contrary, CsgA_CC_ amyloid formation can be started and stopped at any desired time by the addition of a reducing or oxidizing agent (Figure 6). The ability to halt amyloid formation at different points in the process could allow the characterization of amyloid fiber intermediates. Thus the earliest amyloid processes such as the transition of monomers to oligomers or the formation of nucleating species can be better resolved.

After the addition of an oxidizing agent to CsgA_CC_+TCEP, there is no decrease in Th-T fluorescence observed, but instead the Th-T positive species that had formed seem to persist for at least 12 h (Figure 6), suggesting two things. First, during the Th-T growth phase there must be monomers in solution which are still susceptible to forming a disulfide bond that makes them amyloid-incompetent. Second, in a model of amyloid formation wherein the fiber end is in a state of equilibrium with free protein monomers, there would be a k_on_ and k_off_ that described the addition/subtraction of monomers to/from the fiber. If this were the case, then monomers that subtract from the fiber could become susceptible to oxidation. The excess of oxidizing agent would greatly shift the equilibrium in the direction of free, oxidized protein monomers and fibers would begin to unravel at the fiber ends. Because the curve in Figure 6 does not show a time-dependent decrease in Th-T fluorescence, it appears that oxidized CsgA_CC_ amyloid fibers and their fiber ends are stable over at least 12 h.

## Conclusion

In this study, we have shown the ability to tune aggregation of a functional amyloid protein by engineering a disulfide bond into CsgA from *E. coli*. CsgA_CC_ remains in a soluble, non-amyloidogenic state until the disulfide is reduced (Figures 2A, 4). Once reduced, amyloid formation can be manipulated depending on the redox state of the environment (Figure 6). CsgA_CC_ is a tool that makes studying amyloid formation easier and offers researchers extra time and control, two highly valuable resources. Purified CsgA_CC_ samples can now be prepared ahead of time, concentrated, and stored. This method of using disulfide engineering to control amyloid formation could be translated to other functional and human diseases-causing amyloids. Furthermore, the growing field of bio-inspired nanotechnology is capitalizing on the self-assembly of amyloid proteins. CsgA has been shown to have range of uses such as the passage of electric current, the extraction of rare earth metals, and many others (Nguyen et al., 2014; Seker et al., 2017; Tay et al., 2018). The work presented here will allow material scientists to use amyloid fibers to build systems of higher complexity than is currently possible.

## Data Availability Statement

The datasets generated for this study are available on request to the corresponding author.

## Author Contributions

AB and MC contributed to the conception and design of the study. AB, EK, and SJ performed the experiments. AB wrote the manuscript. AB and MC contributed to the editing and revising of the manuscript.

## Conflict of Interest

The authors declare that the research was conducted in the absence of any commercial or financial relationships that could be construed as a potential conflict of interest.
