# Supplementary material for: Tuning Functional Amyloid Formation Through Disulfide Engineering
Source: Front Microbiol. 2020 May 26;11:944. doi: 10.3389/fmicb.2020.00944 (PMC7264389; doi:10.3389/fmicb.2020.00944)
Supplement: Supplementary file 1 [file Data_Sheet_1.docx]

Supplementary Material

# Supplementary Figures and Tables

## Supplementary Tables

**Table 1. Strains used in this study**

| Strains | Relevant Genotype | References |
| --- | --- | --- |
| MC4100 | F- *araD*139 Δ(*argF-lac*) U169 *rpsL*150(*strR*) *relA*1 *fibB*5301 *deoC*1 *ptsF*25 *rbsB* | (Casadaban, 1976) |
| LSR10 | MC4100 Δc*sgA* | (Chapman et al., 2002) |
| MC1061 | *F– araD139 Δ(ara-leu)7696 galE15 galK16 Δ(lac)X74 rpsL (StrR) hsdR2 (rK– mK+) mcrA mcrB1* | (Casadaban and Cohen, 1980) |
| NEB3016 | MiniF *lacI^q^*(Cam^R^) */ fhuA2 lacZ::T7 gene1 [lon] ompT gal sulA11 R(mcr-73::miniTn10--*Tet^S^*)2 [dcm] R(zgb-210::Tn10--*Tet^S^*) endA1 Δ(mcrC-mrr)114::IS10* | New England Biolabs |
| CsgA | NEB 3016 ΔslyD + pET11d-CsgA -sec C-term 6xHis, amp^r^ | This study |
| ALB1 | NEB 3016 ΔslyD + pET11d-CsgA-sec A63C /V140C C-term 6xHis, amp^r^ | This study |
| ALB6 | NEB 3016 Δ*slyD* + pET11d-CsgA -sec A63C C-term 6xHis, amp^r^ | This study |
| ALB9 | NEB 3016 Δ*slyD* + pET11d-CsgA -sec V140C C-term 6xHis, amp^r^ | This study |
| ALB13 | MC4100 Δc*sgA* + pLR5 (PCsgBAC-CsgA), kan^r^ | This study |
| ALB14 | MC4100 Δc*sgA* + pLR2 (PCsgBAC-EV), kan^r^ | This study |
| ALB15 | MC4100 Δc*sgA* + pLR5 (PCsgBAC-CsgA V140C), kan^r^ | This study |
| ALB16 | MC4100 + pLR2 (PCsgBAC-EV), kan^r^ | This study |
| ALB17 | MC4100 Δc*sgA* + pLR5 (PCsgBAC-CsgA A63C V140C), kan^r^ | This study |
| ALB18 | MC4100 Δc*sgA* + pLR5 (PCsgBAC-CsgA A63C), kan^r^ | This study |

**Table 2. Plasmids used in this study**

| Plasmids | Relevant Characteristics | References |
| --- | --- | --- |
| pET11d | IPTG inducible expression vector | New England Biolabs |
| pLR2 | Control vector containing CsgBAC promoter | (Robinson et al., 2006) |
| pLR5 | *csgA* sequence in pLR2 | (Wang et al., 2008) |

**Table 3. Primers used in this study**

| Primers | Primer Sequence (5’→ 3’) | Constructs |
| --- | --- | --- |
| CsgA_A63C | GCACTTGCTCTGCAAACTGATTGCCGTAACTCTGACTTG | CsgA A63C |
| CsgA_A63C_AS | CAAGTCAGAGTTACGGCAATCAGTTTGCAGAGCAAGTGC | CsgA A63C |
| CsgA_V140C | TCCGTCAACGTGACTCAGTGTGGCTTTGGTAACAACGC | CsgA V140C |
| CsgA_V140C_AS | GCGTTGTTACCAAAGCCACACTGAGTCACGTTGACGGA | CsgA V140C |

## Supplementary Figures


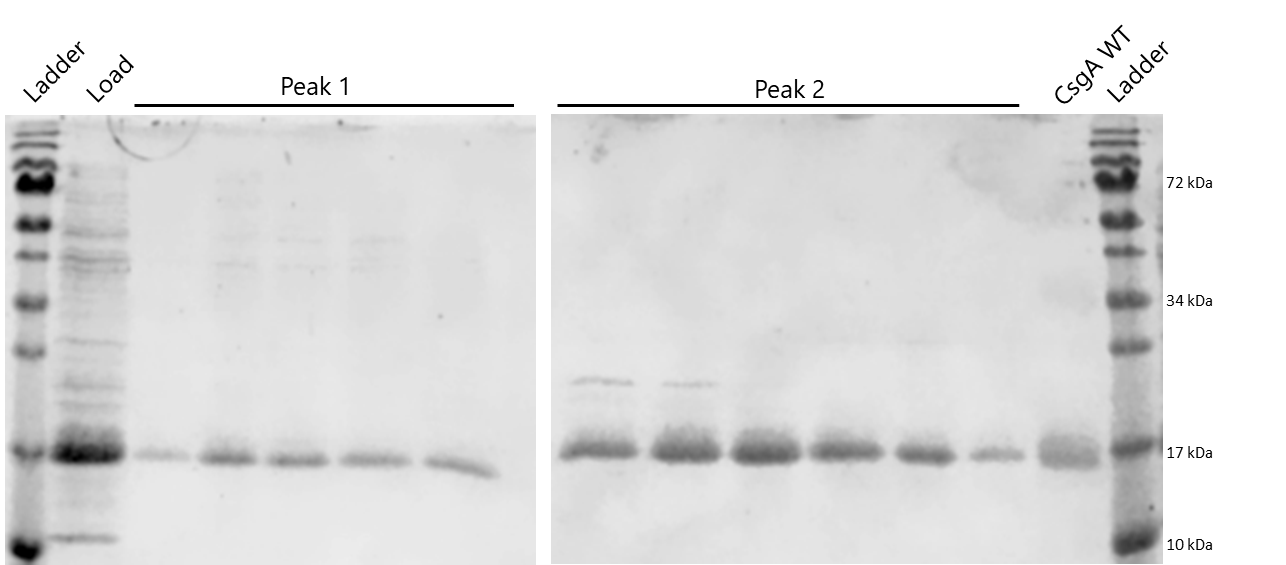


**Supplementary Figure 1. Reducing SDS-PAGE gels showing the protein species in SEC elution fractions corresponding to Peaks 1 and 2.** All samples were prepared in SDS loading dye containing β-mercaptoethanol. The Load lane corresponds to a protein sample taken directly before gel filtration. The elution and CsgA WT samples were taken from the same fractions shown in **Figure 2C**. The gel was stained with Coomassie Blue and destained to visualize protein bands.

**
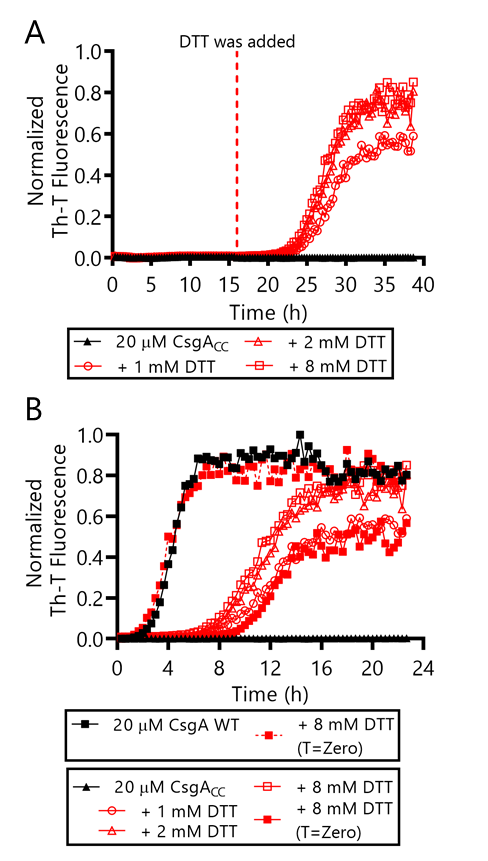
**

**Supplementary Figure 2. Amyloid formation of CsgA_CC_** **monitored by Th-T binding is triggered upon the addition of reducing agent dithiothreitol (DTT). (A)** 20 µM CsgA_CC_ was reduced after a 16 h incubation at room temperature by the addition of DTT to a final concentration stated in the figure legend. **(B)** The same data is represented where T = 0 hours has been adjusted to reflect the addition of DTT, not the beginning of the experiment. There is one supplementary condition shown wherein the CsgA WT protein is also subjected to DTT at 8mM. The only conditions where DTT was added immediately instead of waiting 16 h are marked T=Zero.


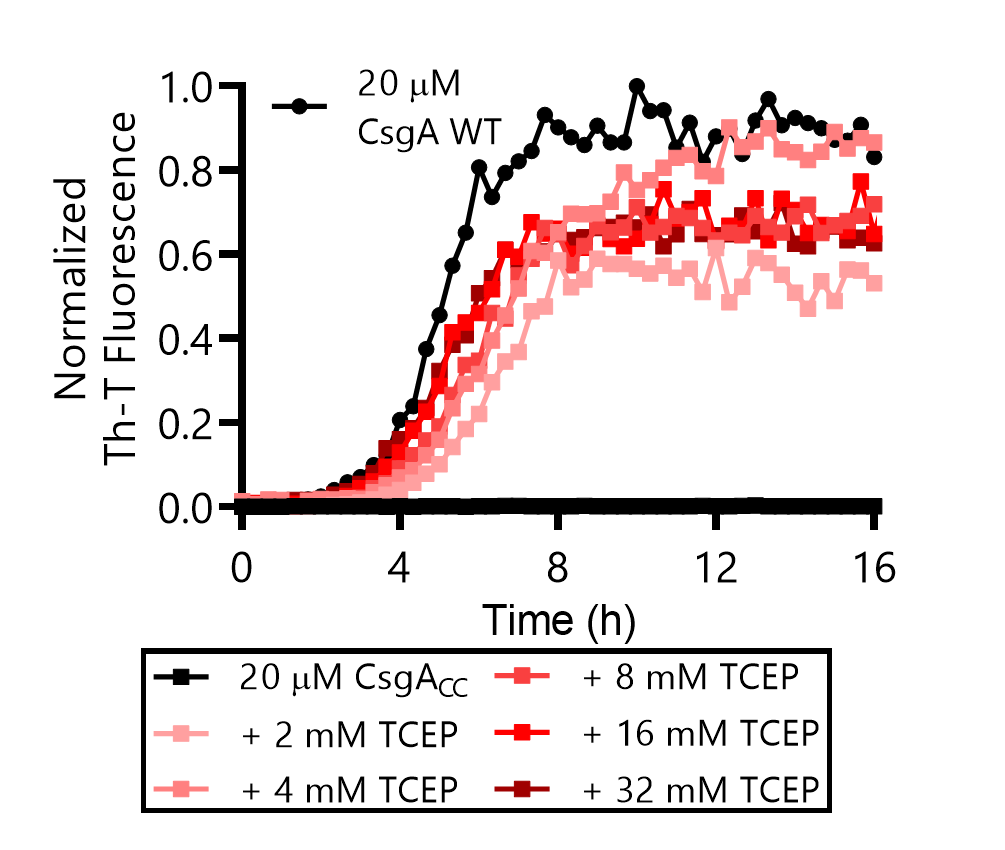


**Supplementary Figure 3.** **Amyloid formation of CsgA_CC_** **monitored by Th-T binding is triggered upon the addition of reducing agent TCEP in the millimolar range.** 20 µM of freshly purified CsgA_CC_ was reduced by increasing amounts of TCEP and incubated at room temperature for 16 hours. The CsgA_CC_ with no reducing agent data are represented by the curve that is mostly superimposed along the x-axis.


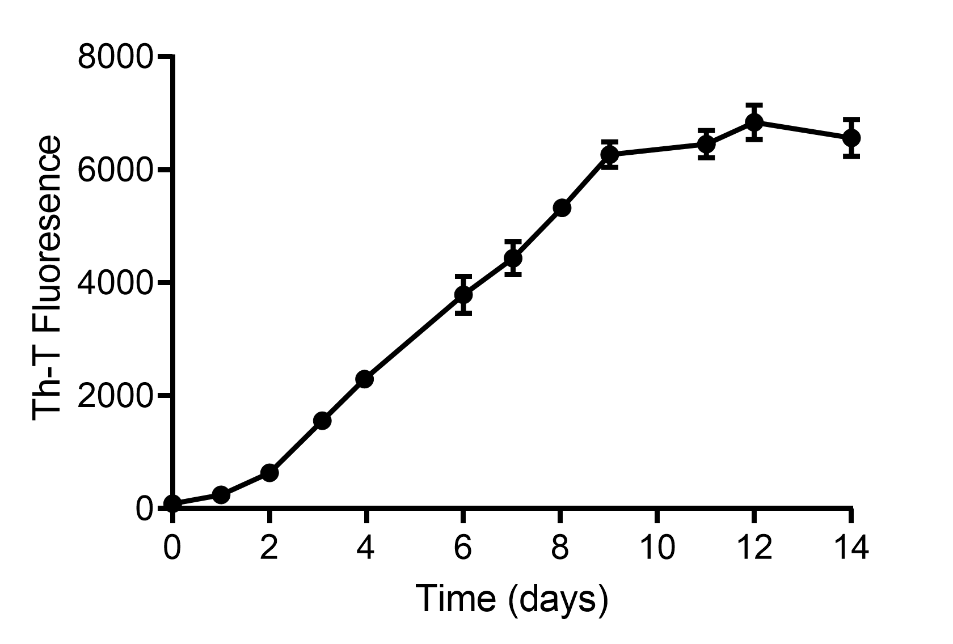


**Supplementary Figure 4.** **Th-T binding assay showing CsgA V140C amyloid formation occur without the addition of reducing agent.** 20 µM of freshly purified and CsgA V140C was incubated at room temperature, without the addition of reducing agent, for 14 days and monitored for amyloid formation using Th-T fluorescence (n = 5). CsgA V140C amyloid formation with and without reducing agent over a 16 h period can be seen in **Figure 5B**.


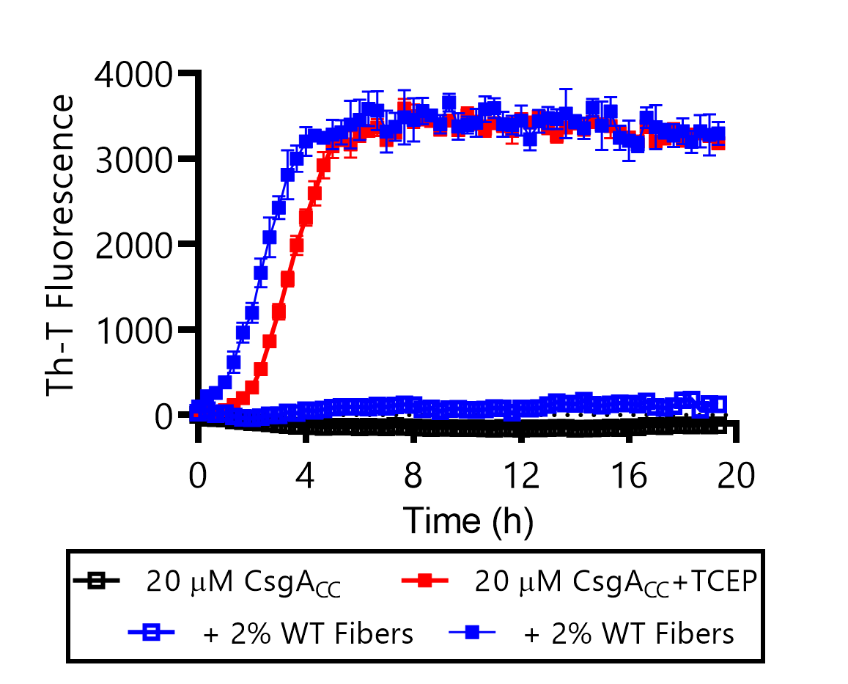


**Supplementary Figure 5. The addition of 2% WT fibers does not cause oxidized CsgA_CC_ to form amyloid.** 20 µM of freshly purified CsgA_CC_ was reduced by TCEP and treated with 2% (w/w) sonicated pre-formed CsgA WT fibers and incubated for 20 h at room temperature. Fibers were also added to a sample of CsgA_CC_ which was never reduced (blue, open squares). The CsgA_CC_ with additives data are represented by the curve that is mostly superimposed along the x-axis.

# References

Casadaban, M. J. (1976). Transposition and fusion of the lac genes to selected promoters in Escherichia coli using bacteriophage lambda and Mu. *J. Mol. Biol.* 104, 541–555. doi:10.1016/0022-2836(76)90119-4.

Casadaban, M. J., and Cohen, S. N. (1980). Analysis of gene control signals by DNA fusion and cloning in Escherichia coli. *J. Mol. Biol.* 138, 179–207. doi:10.1016/0022-2836(80)90283-1.

Chapman, M. R., Robinson, L. S., Pinkner, J. S., Roth, R., Heuser, J., Hammar, M., et al. (2002). Role of Escherichia coli curli operons in directing amyloid fiber formation. *Science (80-. ).* 295, 851–855. doi:10.1126/science.1067484.

Robinson, L. S., Ashman, E. M., Hultgren, S. J., and Chapman, M. R. (2006). Secretion of curli fibre subunits is mediated by the outer membrane-localized CsgG protein. *Mol. Microbiol.* 59, 870–881. doi:10.1111/j.1365-2958.2005.04997.x.

Wang, X., Hammer, N. D., and Chapman, M. R. (2008). The molecular basis of functional bacterial amyloid polymerization and nucleation. *J Biol Chem* 283, 21530–21539. doi:10.1074/jbc.M800466200.
